# Improving Grain Shape of the Rice Variety Exiangsimiao by Introgressing the Long-Grain Allele of *GL7*/*GW7*

**DOI:** 10.3390/ijms27198930

**Published:** 2026-10-08

**Authors:** Hui Hu, Zhilian Qi, Zheqin Wang, Nannan Liu, Junying Xu, Haiyang Liu, Longwei Yang, Jiefeng Jiang, Famao Liang, Xianjin Qiu

**Affiliations:** 1 College of Arts and Sciences, Yangtze University, Jingzhou 434020, China; 13476974813@163.com (H.H.); shuyamino777@163.com (Z.Q.); wang06080715@163.com (Z.W.); rnn070921@163.com (N.L.); 2 Hubei Key Laboratory of Waterlogging Disaster and Agricultural Use of Wetland/Key Laboratory of Green and Efficient Production of Crops in the Middle Reaches of the Yangtze River, Ministry of Agriculture and Rural Affairs (Coconstructed by Ministry and Province), Yangtze University, Jingzhou 434025, China; jyxu@yangtzeu.edu.cn (J.X.); hyliu@yangtzeu.edu.cn (H.L.); ylwei1968@126.com (L.Y.); 3 Ningbo Academy of Agricultural Sciences, Ningbo 315101, China; jiefjiang@126.com; 4 Yichang Academy of Agricultural Sciences, Yichang 443004, China; liangfamao92@163.com

**Keywords:** rice, grain shape, marker-assisted selection, *GL7*/*GW7*

## Abstract

Exiangsimiao (EXSM) is a high-yield and high-quality *indica* variety and restorer line. However, its grains are not sufficiently slender. In this study, the long-grain allele of the grain shape gene *GL7*/*GW7* was introgressed into EXSM via marker-assisted selection combined with backcross breeding, and two improved long-grain lines (EXSM-IL1 and EXSM-IL2) were developed. Their background recovery rates reached 96.70% and 97.50%, respectively. Their grain lengths (GLs) and length-to-width ratios (LWRs) were significantly higher than EXSM. The milling quality, especially the head milled rice rate (HMRR), of both improved lines was significantly lower than EXSM. EXSM-IL1 showed no significant difference in grain yield (GY) to EXSM, although both improved lines had shorter total growth duration (TGD), longer panicle length (PL), and a lower seed setting ratio (SSR) and 1000-grain weight (KGW). Six hybrid combinations derived from the improved lines exhibited longer GL, higher LWR and narrower GW than those of the hybrid combinations derived from EXSM. The TGD and KGW of the six hybrid combinations were significantly lower compared with the hybrids from EXSM. Furthermore, the two improved lines carried 41 favorable genes associated with crucial traits. These results provide the basis for rice grain shape improvement and supply long-grain and high-quality breeding lines and restorer lines for rice production in southern China.

## 1. Introduction

Over the past three decades, with the improvement in people’s living standards, rice grain quality has attracted increasing attention. Improving grain quality has become an equally important breeding objective as yield enhancement in rice breeding programs [1]. Rice appearance and quality directly determines consumers’ preference and thus is particularly critical. It mainly includes grain shape and chalkiness. Grain shape traits encompass grain length (GL), grain width (GW) and the length-to-width ratio (LWR), whereas chalkiness-related traits involve the percentage of grains with chalkiness (PGWC) and the chalkiness degree (DEC). Preferences for rice grain shape vary remarkably across countries and regions. In addition, grain shape affects 1000-grain weight (KGW) and the head milled rice rate (HMRR), thereby exerting influences on grain yield and milling quality [2]. Therefore, cloning genes governing grain shape and adopting molecular breeding to optimize grain shape are of great significance for raising rice yield and improving grain quality.

Rice grain shape is a typical quantitative trait controlled by multiple genes and influenced by environmental factors. With the rapid advances in rice functional genomics and molecular marker technology, geneticists have mapped hundreds of quantitative trait loci (QTLs) regulating grain shape using diverse genetic populations, and approximately 80 grain shape genes have been cloned [3]. Genome-wide association studies have revealed that *GS3* [4], *GS5* [5], *GW5* [6,7] and *GL7*/*GW7* [8,9] are the four major genes for grain shape in rice germplasms [10,11,12,13]. *GS3* encodes the γ subunit of heterotrimeric G protein and mainly regulates GL and the LWR [14]. A mutation from codon TGC (encoding cysteine at the 55th amino acid) to the termination codon TGA in exon 2 leads to premature protein translation and consequently generates the long-grain phenotype [4]. *GS5* encodes a serine carboxypeptidase that primarily controls GW [5]. Two key single-nucleotide polymorphisms (SNPs) in the promoter region of *GS5* cause differential gene expression in young panicles, which contributes to variations in grain size [15]. *GW5* encodes a calmodulin-binding protein [16], and a 1212 bp deletion in its upstream region alters gene expression and ultimately modulates GW [6]. *GL7* encodes a homolog of the *Arabidopsis* LONGIFOLIA protein, which regulates longitudinal cell elongation and affects both GL and GW [9]. Nine haplotypes of *GL7* have been identified in rice germplasms [12]. A 17.1 kb tandem duplication at this locus upregulates the expression of *GL7* and suppresses the expression of adjacent negative regulators, resulting in increased GL and improved grain appearance quality [9]. Moreover, the MYB transcription factor AH2 binds to the promoter of *SLG7* and represses its transcription [17]. *GW8* can also directly bind to the promoter of *GW7* to inhibit gene expression [8]. Among 12 long-grain *indica* rice varieties in southern China, approximately 33% carried the long-grain and narrow-grain alleles of *GS3*, *GS5*, and *GL7*/*GW7* simultaneously [18]. Under the ZS97 genetic background, grain LWRs were significantly increased in the individual introgression lines of *GS3*, *GS5* and *GL7*/*GW7* [11]. Pyramiding of these genes revealed that the LWR of pyramided lines rose as more genes were combined, suggesting additive effects rather than epistatic interactions among these three genes for the grain LWR.

Marker-assisted selection (MAS) enables direct selection of genes associated with target phenotypes and greatly improves breeding efficiency and precision. For example, Liu et al. pyramided *Pi9*, *Pi5* and *Pi54* into Huhan 1S, combined with drought tolerance screening, and developed Huhan 74S, a water-saving and drought-resistant two-line male sterile line with improved blast resistance [19]. Yang et al. pyramided *Pi2*, *Xa23*, *BPH14* and *BPH15* into Feng39S, simultaneously improving its resistance to rice blast, bacterial blight and brown planthopper [20]. Jan et al. introduced the high-temperature tolerance gene *OsHTAS* into HT13 and significantly enhanced its heat tolerance at the heading stage [21]. Using Diantun 502 and Yunhui 290 as parents, Wu et al. integrated conventional crossing with MAS for the soft rice gene *Wx^hp^* and fragrance gene *badh2*, and developed the soft fragrant rice variety Diangu 1893 [22]. Wang et al. introgressed and pyramided *GS3*, *Chalk5* and *Chalk7* into the ZS97 genetic background and successfully improved its appearance quality [11].

In recent years, rising consumer demands for superior grain quality have created a strong market demand for rice with a LWR greater than 3.5 in southern China [23]. However, most rice varieties, especially hybrid rice cultivars, fail to meet this standard. Previous studies indicated that most *indica* varieties in southern China generally carry the *gs3* allele [12]. Introgression of the long-grain allele of *GL7*/*GW7* into these varieties can substantially increase the LWR and improve appearance quality. Nevertheless, few studies have reported molecular breeding for grain shape improvement, and research on grain shape modification under hybrid rice backgrounds remains scarce.

Exiangsimiao (EXSM) is an elite conventional variety with high yield and excellent grain quality. In addition, it is the restorer parent of hybrid combination Zhenliangyou Exiang Simiao and Taiyou Exiangsimiao. However, its LWR is only 3.5, and the grains are not sufficiently slender. In the present study, MAS was adopted to introgress the long-grain allele of *GL7*/*GW7* into EXSM. We evaluated the effects of *GL7*/*GW7* introgression on grain shape, chalkiness, milling quality and yield-related traits. Furthermore, testcrosses were performed between the improved lines and various male sterile lines to assess the gene effects under hybrid rice backgrounds. The results will provide a theoretical foundation for molecular improvement of grain shape in *indica* and supply valuable germplasm for breeding long-grain rice varieties.

## 2. Results

### 2.1. Introgression of Long-Grain Allele of Grain Shape Gene *GL7*/*GW7* by MAS

**Figure 1:**
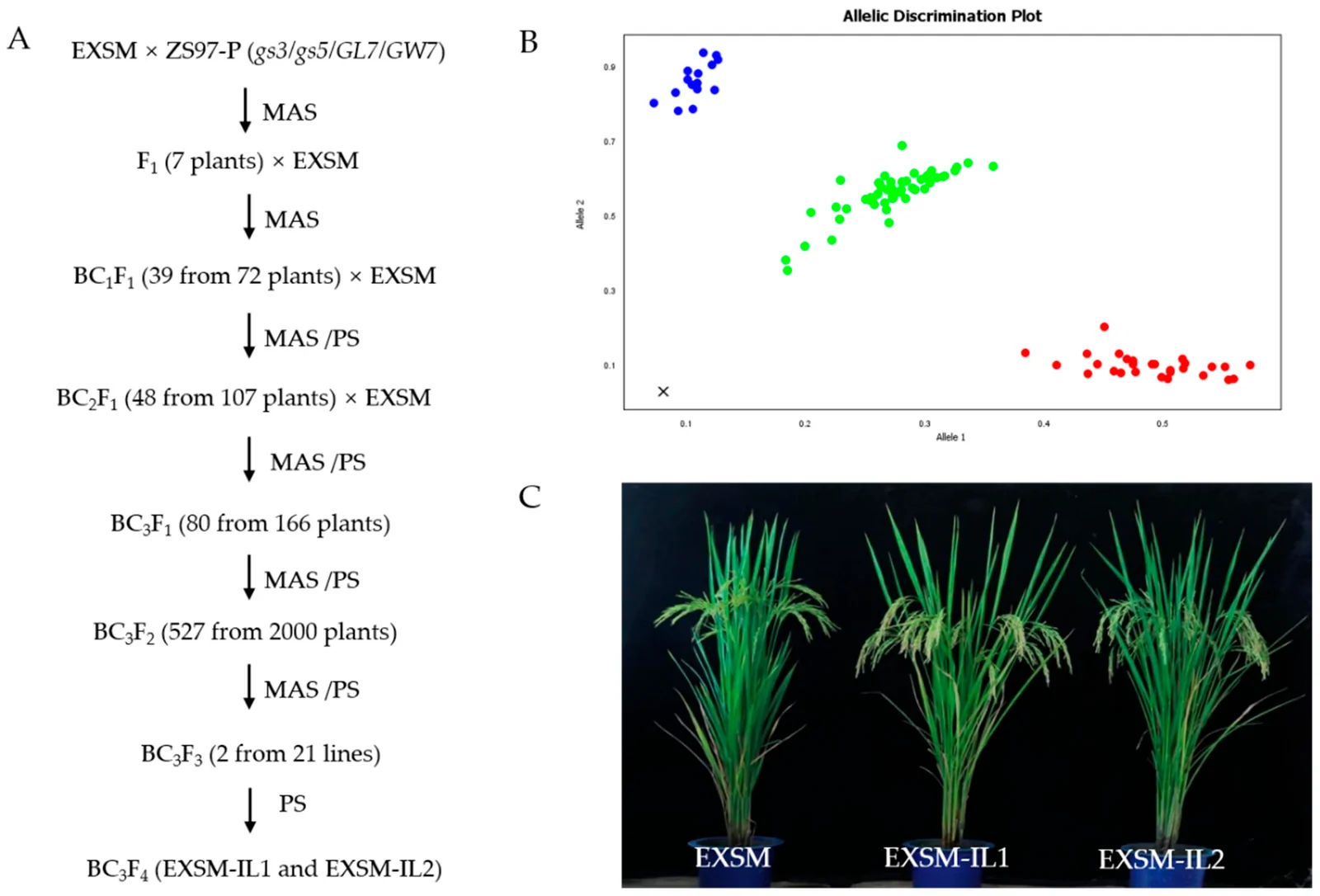
Scheme showing introgression of long-grain allele of grain shape gene *GL7*/*GW7*. (**A**), the introgression scheme. PS, phenotype selection. (**B**), genotyping of *GL7*/*GW7* in the BC_3_F_2_ generation. Blue represents EXSM homozygous genotype, red represents ZS97-P homozygous genotypes, green represents heterozygous genotype; ×, negative control. (**C**), plant type of the two improved lines at mature stage.

As shown in Figure 1A, EXSM was crossed as the female parent with ZS97-P as the male parent. Twelve F_1_ plants were grown, and the genotype of *GL7*/*GW7* of each individual was detected using the K-Chalk7 marker. Seven true F_1_ hybrids were identified and backcrossed with EXSM. Seventy-two BC_1_F_1_ individuals were planted, among which 39 plants carrying *GL7*/*GW7* were identified via K-Chalk7 genotyping. One individual with agronomic traits similar to EXSM was selected for further backcrossing. A total of 107 BC_2_F_1_ plants were planted, and 48 individuals harboring *GL7*/*GW7* were screened by K-Chalk7. Two plants exhibiting agronomic performance similar to EXSM were chosen to conduct backcrossing. One hundred and sixty-six BC_3_F_1_ individuals were grown, and 80 plants carrying *GL7*/*GW7* were identified using K-Chalk7. Three individuals with agronomic traits largely consistent with EXSM were selected. A population consisting of 2000 BC_3_F_2_ plants was planted, and 527 individuals homozygous for the introgressed *GL7*/*GW7* allele were identified by K-Chalk7 (Figure 1B). Twenty-one individuals with superior comprehensive agronomic traits were selected. Twenty-one BC_3_F_3_ lines were planted, from which two elite individuals with excellent overall performance were selected. Two BC_3_F_4_ lines were grown to evaluate line stability, and these two lines were finally designated EXSM-IL1 and EXSM-IL2 (Figure 1C).

### 2.2. Genetic Background Examination of the Two Improved Lines

**Figure 2:**
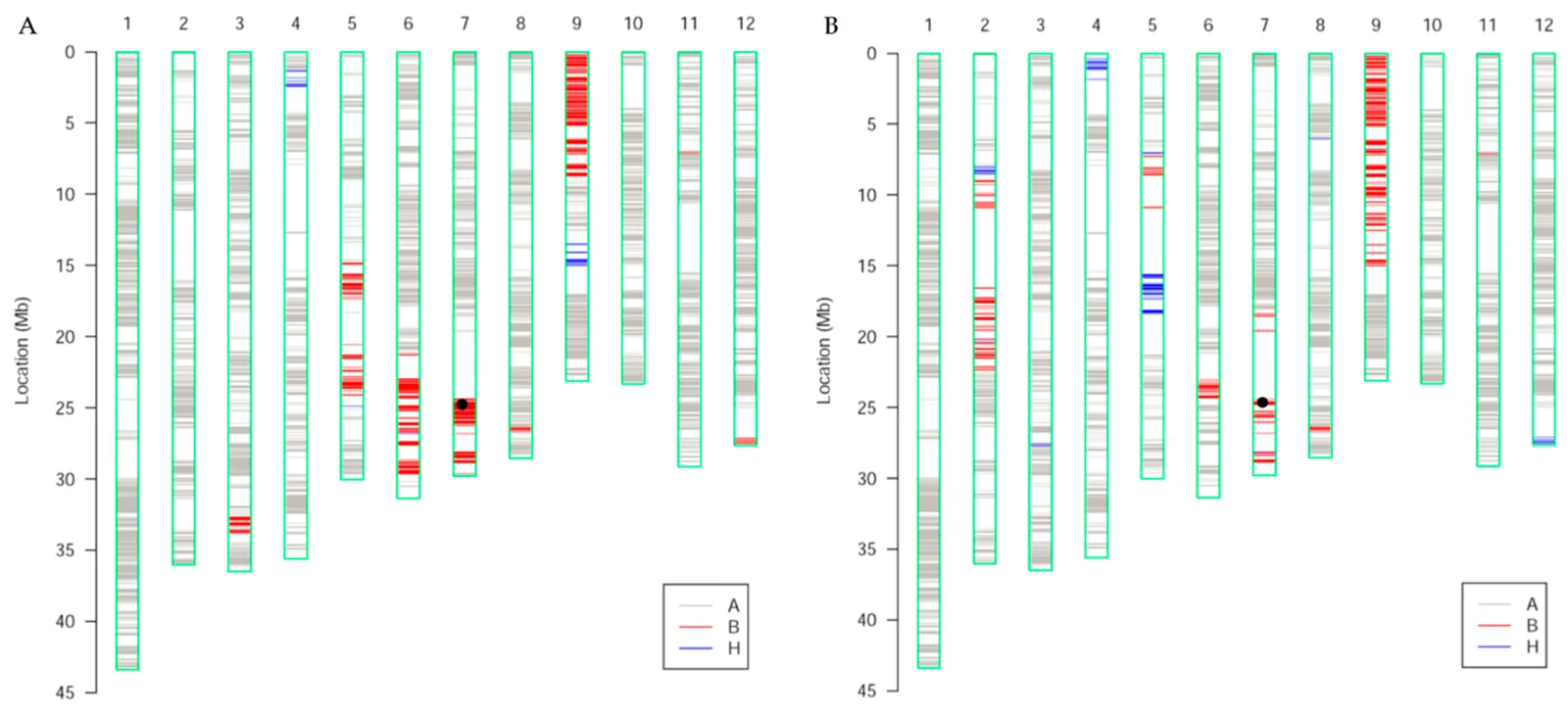
Haplotype maps of genetic background profiling using RICE40K array. (**A**), EXSM-IL1; (**B**), EXSM-IL2. Gray represents EXSM homozygous genotype, red represents ZS97-P homozygous genotypes, blue represents heterozygous genotype. The dots represent the position of *GL7*/*GW7* on chromosome 7.

Genome-wide genetic background of the two improved lines was examined using the RICE40K SNP array, a whole-genome SNP array. The RICE40K array contains approximately 30,000 SNP markers, among which 7917 markers exhibited polymorphisms between EXSM and ZS97-P. Based on the proportion of polymorphic markers, the background recovery rates of EXSM-IL1 and EXSM-IL2 reached 96.70% and 97.50%, respectively (Figure 2). Owing to the introgression of *GL7*/*GW7*, both improved lines carried a large introgressed segment on the long arm of chromosome 7 (10.00 Mb from 19.63 Mb to the end in EXSM-1 and 11.24 Mb from 18.40 Mb to the end in EXSM-2). In addition, EXSM-IL1 possessed three large introgressed segments on chromosomes 5, 6 and 9, while EXSM-IL2 contained three large introgressed segments on chromosomes 2, 5 and 9. Nevertheless, heterozygous segments were detected at other chromosomal regions in both lines. Specifically, heterozygous segments were found on chromosomes 2 and 9 in EXSM-IL1, whereas heterozygous segments were located on chromosomes 2, 4, 5 and 12 in EXSM-IL2.

### 2.3. Appearance Quality and Milling Quality Analysis of the Two Improved Lines

**Figure 3:**
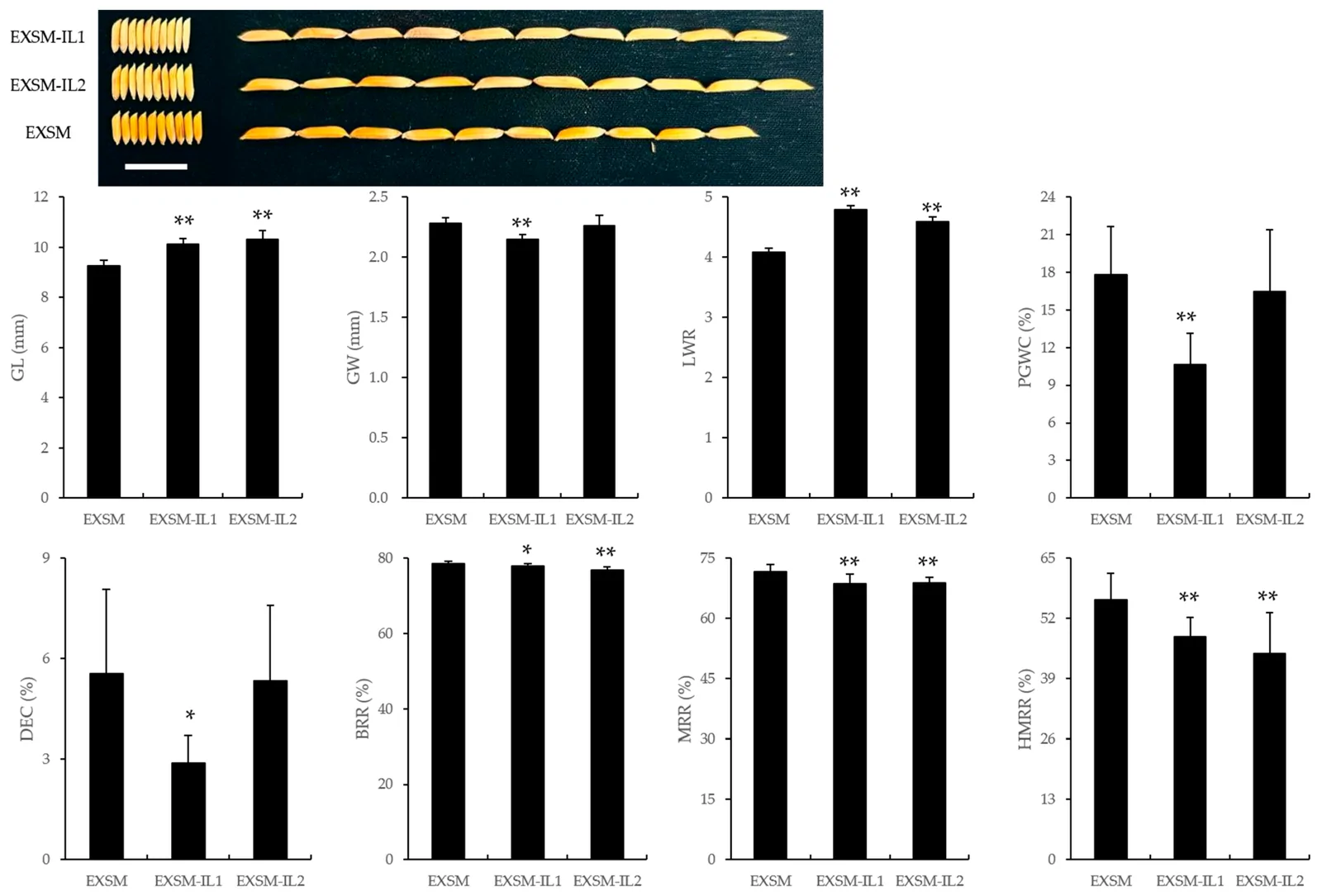
Appearance quality and milling quality of the two improved lines. * and ** represent significance level of *p* < 0.05 and 0.01, respectively. Scale bar, 1 cm.

The appearance and milling quality of the two improved lines are presented in Figure 3. The GL of EXSM-IL1 and EXSM-IL2 were 10.13 mm and 10.30 mm respectively, which were significantly longer than the 9.26 mm of EXSM. Their GWs were 2.15 mm and 2.26 mm. Notably, EXSM-IL1 exhibited a significantly narrower GW than EXSM (2.28 mm). Consequently, the LWR of the two improved lines reached 4.79 and 4.59, both significantly higher than EXSM. Meanwhile, the PGWC and DEC of EXSM-IL1 were 10.65% and 2.88%, respectively, which were markedly lower than those of EXSM and EXSM-IL2.

Nevertheless, the milling quality of both improved lines decreased significantly. For instance, their BRR values were 77.98% and 76.94%, slightly lower than the 78.49% of EXSM, and the differences were statistically significant. The MRR values of the two lines were 68.68% and 68.92%, both extremely significantly lower than those of EXSM. More importantly, HMRR values of EXSM-IL1 and EXSM-IL2 were 48.07% and 44.46%, substantially lower than the 56.02% of EXSM.

These results demonstrate that the introgression of *GL7*/*GW7* effectively increased GL and the LWR, achieving successful grain shape improvement.

### 2.4. Yield-Related Trait Analysis of the Two Improved Lines

Significant differences in several yield-related traits were detected between the two improved lines and EXSM (Table 1). The TGD of EXSM-IL1 and EXSM-IL2 were 122.55 d and 123.50 d, respectively, approximately 8 days shorter than that of EXSM. The PLs of the two improved lines were 28.05 cm and 28.21 cm, around 1.0 cm longer than EXSM. The SN and GN of EXSM-IL1 were 209.59 and 165.38, which were extremely significantly higher than those of EXSM (168.93 and 129.67). Nevertheless, the SSR of EXSM-IL1 and EXSM-IL2 was 73.15% and 69.60%, both extremely significantly lower than the 77.75% of EXSM. The KGWs of the two improved lines were 19.71 g and 20.54 g, significantly lower than EXSM (21.55 g). Ultimately, no significant difference in GY was observed between EXSM-IL1 and EXSM, whereas EXSM-IL2 exhibited a significantly lower GY than EXSM.

### 2.5. Analysis of the Yield-Related Traits, Milling Quality and Appearance Quality for the Hybrid Combinations Derived from the Two Improved Lines and Three Male Sterile Lines

Nine hybrid combinations were generated by crossing EXSM and its two improved lines with three male sterile lines (Table 2). The three male sterile lines were genotyped using three markers (K-GS3 for *GS3* [11], GS5-1 and GS5-2 for *GS5* [18], K-Chalk7 for *GL7*/*GW7*), and all of their alleles were a long-grain allele for *GS3*, narrow allele for *GS5* and short allele for *GL7*/*GW7*.

In terms of yield-related traits, the TGD of six combinations derived from the two improved lines and three male sterile lines ranged from 127.67 d to 132.22 d, which were all significantly shorter than those of the combinations of EXSM with the three male sterile lines (133.33 d~136.67 d). Except for Q9311A/EXSM-IL2, the average KGW of the remaining hybrid combinations was 25.36 g, significantly lower than that of the hybrids between EXSM and the three male sterile lines (26.72 g). The PH of hybrids between the two improved lines and Q211S were significantly lower than Q211S/EXSM (128.67 cm and 127.67 cm vs. 133.33 cm). The PBN of Q211S/EXSM-IL1 was significantly less than that of Q211S/EXSM (14.67 vs. 15.53). Z9311S/EXSM-IL1 exhibited a significantly longer PL than Z9311S/EXSM (30.42 cm vs. 29.25 cm). Z9311S/EXSM-IL2 had significantly more SN (196.90 vs. 152.46) and a significantly lower SSR (64.01% vs. 70.68%) relative to Z9311S/EXSM. Meanwhile, the GN of the two hybrids derived from the two improved lines and Z9311S was significantly higher than that of Z9311S/EXSM (149.98 and 158.77 vs. 137.68). For Q9311A/EXSM-IL1, PH, PBN and SSR were all significantly lower than those of Q9311A/EXSM (121.00 cm vs. 124.67 cm; 13.73 vs. 15.20; 63.57% vs. 70.45%). The PL of Q9311A/EXSM-IL2 was significantly shorter than that of Q9311A/EXSM (26.87 cm vs. 28.56 cm). Ultimately, no significant difference in grain yield per plant was observed between the hybrid combinations of the two improved lines with three male sterile lines and those of EXSM with the three male sterile lines. These results suggested that although introgression of *GL7*/*GW7* may alter several yield-related traits under hybrid genetic backgrounds, it does not affect the final GY.

With respect to appearance and milling quality, the GL of six combinations derived from the two improved lines and three male sterile lines varied from 9.76 mm to 10.27 mm, all significantly longer than those of hybrids between EXSM and the three male sterile lines (9.43 mm~9.84 mm). Their GW ranged from 2.27 mm to 2.59 mm, significantly narrower than those of EXSM-derived hybrids (2.70 mm~2.73 mm). Consequently, their average LWR reached 4.18, which was extremely significantly greater than that of the EXSM hybrid combinations. The findings demonstrated that the introgression of *GL7*/*GW7* could increase GL and the LWR and reduce GW even under hybrid backgrounds. The MRR of the six combinations ranged from 70.78% to 72.84%, significantly lower than that of the EXSM hybrid combinations (73.94%~73.96%). Q211S/EXSM-IL2 showed significantly lower PGWC and HMRR than Q211S/EXSM (22.00% vs. 27.80%; 38.94% vs. 55.20%). The BRR of Z9311S/EXSM-IL2 was significantly lower than that of Z9311S/EXSM (78.35% vs. 80.49%). Q9311A/EXSM-IL2 exhibited a significantly reduced PGWC and HMRR compared with Q9311A/EXSM (13.60% vs. 17.20%; 47.82% vs. 53.91%).

### 2.6. Profiling of Favorable Alleles in the Two Improved Lines

We performed favorable gene analysis of the two parents and two improved lines using the RICE40K SNP array (Table 3). Approximately 140 genes were surveyed by the RICE40K platform. Among them, EXSM, ZS97-P, EXSM-IL1 and EXSM-IL2 carried 41, 23, 41 and 41 favorable genes, respectively. Both EXSM-IL1 and EXSM-IL2 harbored nine genes regulating SN and grain shape; seven heading date-related genes; four plant architecture genes; one fertility-related gene; two genes associated with superior eating quality; eight genes conferring resistance to rice blast, bacterial blight and brown planthopper; six genes for abiotic stress tolerance; one gene enhancing ratooning ability; and two genes improving fertilizer use efficiency. These genes endow the two improved lines with the potential for desirable agronomic characteristics including high yield, good grain quality, and resistance to pests and diseases.

The sets of favorable genes carried by the two improved lines were not identical. EXSM-IL1 contains the rice blast resistance gene *Pi5*, which confers stronger blast resistance. EXSM-IL2 carries BG2, enabling the formation of larger grains.

## 3. Discussion

### 3.1. The Effect of *GL7*/*GW7* on the Improved Lines and Their Derived Hybrids

*GS3*, *GS5* and *GL7*/*GW7* are three core genes for rice grain shape improvement. Gene profiling of recipient and donor parents was conducted using the RICE40K genome-wide SNP array. The results showed that both EXSM and ZS97-P carried favorable alleles of *GS3* and *GS5* (Table 3). Therefore, only the introgression of *GL7*/*GW7* from ZS97-P into EXSM was required to substantially modify grain shape. In the present study, *GL7*/*GW7* was successfully introgressed into EXSM after three backcrosses and four selfing generations. The GL of EXSM-IL1 and EXSM-IL2 were 10.13 mm and 10.30 mm, with an LWR of 4.79 and 4.59, respectively, all significantly greater than those of the recurrent parent EXSM. Moreover, the grain shape of hybrid combinations derived from the two improved lines and three male sterile lines was significantly superior to that of hybrids between EXSM and the three male sterile lines, indicating successful grain shape improvement in both homozygous genetic background and hybrid genetic background. In recent years, long-grain fragrant rice varieties have gained great popularity in the Yangtze River Basin of China, bringing farmers substantially higher income compared with conventional rice cultivars. For instance, in 2025, the average grain price of long-grain fragrant rice in Hubei Province was 0.6 CNY per kilogram higher than that of ordinary rice, corresponding to an additional income of approximately 5000 CNY per hectare. Moreover, the consumer market for long-grain fragrant rice keeps expanding across southern China, and consumers show an increasing preference for this type of rice. Therefore, the improved lines developed and their derived hybrid combinations are a good match for the current demand for high-quality rice in the Yangtze River Basin. They would play a vital role in raising farmers’ planting income and satisfying consumer requirements.

Milling quality, especially the HMRR, decreased significantly in both improved lines, demonstrating that introgression of *GL7*/*GW7* impairs milling quality of rice. This finding is consistent with previous reports that the LWR is significantly negatively correlated with the HMRR [2]. In addition, the Yangtze River Basin suffered extreme high-temperature stress during rice heading in 2025 [24]. The SSRs of improved lines were significantly lower than that of recurrent parent EXSM, suggesting that introgression of *GL7*/*GW7* may weaken the high-temperature tolerance of the improved lines. Fortunately, no significant reduction in the HMRR and SSR was observed in most hybrid combinations, implying that the genetic background of male sterile lines can compensate for these negative effects induced by *GL7*/*GW7*.

The heading date of the two improved lines and their hybrid combinations was significantly earlier than that of EXSM and its corresponding hybrids (Table 1 and Table 2). In terms of the identification of favorable genes among the detected genes associated with the heading date, the haplotype of *Ghd7.1* in the two improved lines differed from EXSM and was identical to donor parent ZS97-P (Table 3). *Ghd7.1* is located at 29.62 Mb on rice chromosome 7 [25], and is 4.95 Mb away from *GL7*/*GW7*. Genetic background analysis confirmed a large introgression segment on the long arm of chromosome 7 in both improved lines (Figure 2). Consequently, *Ghd7.1* from ZS97-P was co-introgressed together with *GL7*/*GW7*, ultimately shortening the TGD of the improved lines. Similar unfavorable phenotypic changes caused by linkage drag have been documented in previous research. For example, when Zeng et al. introgressed *Pi2* into Changnongjing 1, a large segment spanning 1.32–25.11 Mb on chromosome 6 was introgressed simultaneously, leading to remarkable changes in eating quality and significant yield reduction in the improved line [26]. Hence, when improving target traits via MAS, breeders should evaluate whether unfavorable genes are tightly linked to the target gene. If linkage drag exists, flanking markers should be adopted to screen recombinant individuals during backcrossing to eliminate undesirable linked genes.

The results showed a significant quality–yield trade-off in the improved lines (improved grain shape accompanied by a reduced HMRR and SSR). To solve this problem, we present two strategies. Firstly, because *Ghd7.1* is only 4.95 Mb away from *GL7*/*GW7*, we could reduce the size of the introgressed segments through backcrossing and other approaches so as to mitigate linkage drag. Secondly, we could introgress favorable alleles of some important genes for GY and milling quality into the improved lines to break the quality–yield trade-off, such as *IPA1* [27], *GY3* [28], *GW2* [29] and *Chalk5* [30]. However, very few genes for the HMRR and SSR have been cloned previously, and we should develop a lot of segregating populations derived from the improved lines to identify QTLs for the two traits, and introgress them into the improved lines to increase their HMRR and SSR.

### 3.2. Efficiency of the Backcross via MAS

Phenotypic selection via conventional breeding remains the most prevalent strategy in rice breeding, and has successfully led to the breeding of numerous elite rice varieties. Rice grain shape is a typical quantitative trait with high genetic heritability [2]. Therefore, varieties with a large LWR can be bred through conventional breeding. Nevertheless, most long-grain rice varieties developed by conventional breeding generally exhibit superior grain quality accompanied by low grain yield [31]. This is mainly attributed to the complexity of conventional breeding. Breeders simultaneously select multiple genes governing yield and quality, which imposes great difficulties and requires longer time. Furthermore, *GL7*/*GW7* exhibits semi-dominant inheritance. Heterozygous genotypes present a relatively low LWR, further limiting selection efficiency. MAS combined with backcross breeding enables rapid improvement in existing elite varieties. Theoretically, its selection efficiency is three times higher than that of conventional breeding, while other agronomic traits of the recurrent parent are largely maintained. In this study, *GL7*/*GW7* was introgressed into EXSM, which not only significantly improved the grain shape of EXSM, but also exerted no adverse effect on the grain yield of EXSM itself and its derived hybrid combinations, achieving high breeding efficiency and a high success rate.

Genetic background analysis of EXSM-IL1 and EXSM-IL2 was performed using the RICE40K genome-wide SNP array. The results revealed that after three rounds of backcrossing and four generations of selfing, the background recovery rates of the two improved lines reached 96.70% and 97.50%, respectively (Figure 2). Generally, larger population sizes together with foreground and background selection are required to increase background recovery rates in backcross breeding. For instance, Suh et al. improved bacterial blight resistance of Mangeumbyeo via backcross breeding without marker-assisted background selection, and only obtained a background recovery rate of 92.1% after three backcrosses [32]. Yang pyramided *Pi2*, *Xa23*, *BPH14* and *BPH15* into Feng39S [20]. Owing to simultaneous foreground and background selection using molecular markers, four near-isogenic lines achieved high recovery rates ranging from 98.77% to 99.16% after three and four backcross generations. However, continuous background selection in each generation substantially increases breeding costs. Accordingly, the combination of MAS and PS can rapidly elevate background recovery rates while significantly reducing costs. Zeng et al. introgressed *Pi2* into Changnongjing 1 to improve blast resistance [26]. By integrating MAS and PS, the background recovery rate reached 94.36% at the BC_2_ generation [26]. In the present study, after introgression of *GL7*/*GW7* into EXSM, the recovery rates of the two improved lines at the BC_3_ generation (96.70% and 97.50%) were markedly higher than the theoretical value (93.75%). This strategy achieves favorable selection outcomes at low cost, representing an economical and efficient technical approach.

### 3.3. Detection of Favorable Genes in the Improved Lines

Genome-wide analysis using the RICE40K SNP array revealed that each improved line carried 41 favorable alleles. Apart from genes conferring high yield and ideal plant architecture, both lines harbored six rice blast resistance genes, two bacterial blight resistance genes and one brown planthopper resistance gene, endowing them with potential resistance to these three major pests and diseases. Notably, both lines possess *Pi2* (from C101A51) and *Pi-ta* (from Pai-kan-tao), two dominant resistance genes widely deployed in rice production across the Yangtze River Basin and South China, contributing to strong blast resistance [33]. Furthermore, both improved lines contain *Wx^b^* and *badh2*, which confer excellent eating quality and strong fragrance. Nine additional genes related to abiotic stress tolerance and nutrient use efficiency enhance resistance to saline–alkali conditions, drought, high aluminum, high sodium and low temperature, as well as nitrogen and potassium utilization efficiency. These genes endow the two improved lines with the potential for desirable agronomic characteristics. In summary, these two improved lines are long-grain and high-quality breeding lines and restorer lines, which serve as valuable germplasm and hybrid rice parents for rice production in southern China.

### 3.4. Limitations of the Present Study

The Yangtze River Basin in China frequently experiences extreme high-temperature weather during summer, which poses a serious threat to middle-season rice production. In the present study, the two improved lines exhibited a significantly reduced SSR and HMRR in 2025, suggesting that the introgression of *GL7*/*GW7* may lead to the deterioration in important agronomic traits under extreme high-temperature conditions. Therefore, breeders should be aware of such negative effects when utilizing this gene in the future, and should particularly consider whether it can be reliably deployed for genetic improvement under extreme high-temperature conditions. However, such changes in these two traits were not observed in previous reports, and the improved lines in this study were not single-segment introgression lines. Accordingly, it cannot be concluded that the reductions in these two traits were directly caused by the introgression of this gene. To clarify this issue, segregating populations should be constructed, and a conventional QTL mapping strategy can be used to determine whether the decreases in the SSR and HMRR are attributable to the introgression of *GL7*/*GW7*.

Agronomic traits of the improved lines and their derived hybrids were evaluated only within a single year in this study, especially in the extreme high-temperature condition in 2025. Due to confounding effects from extreme high-temperature conditions, certain traits might show aberrant performance, potentially leading to less-reliable results. Therefore, continuous multi-year evaluations are still required to minimize the impacts of environmental variation on the results.

Heterozygous segments were detected at other chromosomal regions in both lines by genome-wide analysis using the RICE40K SNP array. These heterozygous loci may lead to minor phenotypic variation among different individual plants within the improved lines. Further crossing is required to homozygize these heterozygous segments in subsequent breeding work.

## 4. Materials and Methods

### 4.1. Plant Materials

EXSM was used as the recipient parent in this study, and the donor parent was an improved line at ZS97 genetic background (ZS97-P, contains *gs3*, *gs5* and long-grain allele of *GL7*/*GW7*) [11]. In addition, two two-line male sterile lines, Quan 211S and Zhen 9311S (Q211S and Z9311S), and one three-line male sterile line, Quan9311A (Q9311A), were used as female parents to conduct testcrosses with EXSM and the improved lines.

### 4.2. DNA Extraction, Molecular Markers and PCR

Genomic DNA was isolated from young plant leaves by the CTAB method [2]. The Kompetitive Allele Specific (KASP) marker K-Chalk7 was applied for genotyping of *GL7*/*GW7* [11]. The marker sequence is Chalk7-fam-F: 5′-gaaggtgaccaagttcatgctAAGCTGCAGGCCAGAGATGCGT-3′, chalk7-hex-F: 5′-gaaggtcggagtcaacggattAAGCTGCAGGCCAGAGATGCGC-3′, Chalk7-R: 5′-CTGGTCTCATGCTCGCCAATGCAA-3′. The KASP marker was located in the third exon of *GL7*/*GW7*. The KASP reaction mixture contained 5 μL DNA (~50 ng/μL), 0.15 μL primer mix and 5 μL 2 × KASP Master Mix (KBS-1016-002, LGC, Hoddeston, UK). The PCR program included an initial incubation at 95 °C for 15 min, 10 touchdown cycles (95 °C for 20 s; annealing starting at 61 °C, decreasing by 0.6 °C each cycle for 1 min), and 28 subsequent cycles at 95 °C for 20 s and 55 °C for 1 min. Genotyping of PCR products was performed using the RT-PCR system (CFX-96, BioRad^®^, Hercules, CA, USA).

### 4.3. Genetic Background Analysis and Favorable Gene Identification

RICE40K, a whole-genome SNP array, was used to analyze the genetic background and identify favorable alleles of the improved lines [20]. The RICE40K array contains approximately 30,000 nucleic acid probes, including functional SNP probes corresponding to about 140 genes. The array is based on nucleic acid hybridization and complementary base pairing. Large numbers of probes with known sequences are immobilized at high density on a silicon wafer and hybridized with fluorescently labeled test samples; by detecting the intensity and distribution of the hybridization signals, the genotype of each sample can be determined in a high-throughput, qualitative manner. Young leaves were sampled from around 20 individual plants, and genomic DNA was extracted using a DNA extraction kit (DP305, Tiangen Biotech (Beijing) Co., Ltd., Beijing, China). DNA amplification, fragmentation, chip hybridization, single base extension, staining and scanning were conducted on the genotyping platform of Wuhan Greenfafa Institute of Novel Genechip R&D Co., Ltd., Wuhan, China, Subsequent genetic background analysis and identification of favorable genes were implemented for the improved lines.

### 4.4. Field Experiment

During the summer of 2024, EXSM, improved lines and three male sterile lines were planted on the experimental farm of Yangtze University in Jingzhou, Hubei (30.18° N, 112.15° E), with 30 plants for each line. The improved lines and EXSM were testcrossed with the three male sterile lines respectively, and more than 200 hybrid seeds were obtained from each combination. Meanwhile, EXSM and improved lines were bulk-harvested per line at a mature stage.

In the summer of 2025, EXSM, improved lines and their hybrid combinations were planted on the experimental farm of Yangtze University. The soil at the experimental site had a pH of 6.5, a total nitrogen content of 2.36 g/kg, a total phosphorus content of 0.57 g/kg, an alkali-hydrolyzable nitrogen content of 93.6 mg/kg, an available phosphorus content of 48.3 mg/kg, and an available potassium content of 121.6 mg/kg. In the summer of 2025, seeds were sown on May 7 and seedlings were transplanted on June 11. Before transplanting, 450 kg/ha of compound fertilizer (15-15-15) was applied as basal fertilizer, and 150 kg/ha of urea was applied as topdressing 5 days after transplanting. Shallow water irrigation was maintained throughout the entire growth period of the rice crop. Insecticides were applied three times during the whole growth period to control stem borers and brown planthoppers, and a herbicide was applied once for weed control. In the summer of 2025, the Jingzhou region experienced extreme high temperatures, with maximum daily temperatures exceeding 35 °C on more than 30 days during July–August. Each line was planted in 5 rows with 10 plants per row with two replications under a randomized block design. The planting density was set at 20 cm × 20 cm.

### 4.5. Yield-Related Trait Evaluation

The yield-related traits were evaluated according to the method described by Xu et al. with some minor modifications [34]. At the heading stage, 10 plants were chosen to investigate the heading date, starting from the third plant in the second and third rows, with five consecutive plants used. The plant height (PH) of these 10 plants was measured during grain filling. Total growth duration (TGD) was the time between seedling and maturity. Plants were harvested individually at a mature stage for yield component testing. Panicle number (PN) was counted as panicle numbers greater than five filled grains. Panicle length (PL) was the distance from panicle neck to panicle tip of three randomly chosen panicles. Primary branch number (PBN) was the average count of primary branches on three sampled panicles. Spikelet number per panicle (SN) was calculated as total spikelets per plant divided by PN. Grain number per panicle (GN) was calculated as total filled grains per plant divided by PN. Seed setting ratio (SSR) was calculated as GN/SN × 100%. A total of 500 fully plump grains were bulked and weighed, and KGW was calculated by doubling this value. Yield per plant (GY) was the total weight of all filled grains harvested from each plant.

### 4.6. Appearance Quality and Milling Quality Measurement

After the evaluation of yield-related traits for each individual plant, harvested grains were stored at room temperature for three months to measure milling quality and appearance quality.

Ten randomly selected paddy grains were arranged end-to-end in a straight line, and the total length of the 10 grains was measured. GL was equal to the total length divided by the number of tested grains. Another ten randomly selected paddy grains were aligned side-by-side at their widest positions, and the total width of the 10 grains was measured. GW was calculated as the total width divided by the number of tested grains. The LWR was GL divided by GW.

The quality traits were evaluated according to the method described by Emelin et al. with some minor modifications [2]. Twenty grams of plump seeds were weighed and dehulled using a husker (JMWT12, Beijing Dongfu Jiuheng Instrument Technology Co., Ltd., Beijing, China) to produce brown rice, and the weight of brown rice was recorded. Brown rice rate (BRR) was calculated as brown rice weight/rough rice weight × 100%. Subsequently, all brown rice was milled into polished rice with a rice polisher (JMWT12, Beijing Dongfu Jiuheng Instrument Technology Co., Ltd., Beijing, China), and the weight of polished rice was measured. Milled rice rate (MRR) was calculated as polished rice weight/rough rice weight × 100%. A broken rice separator (FOS, Hangzhou Daji Optoelectronic Instrument Co., Ltd., Hangzhou, China) was used to separate head rice from broken rice, followed by weighing the head rice. Head milled rice rate (HMRR) was calculated as head rice weight/rough rice weight × 100%.

One hundred head milled rice grains were randomly selected to count grains containing chalkiness. PGWC was calculated as (number of chalky grains/total tested grains) × 100%. Ten chalky grains were randomly chosen to measure the ratio of chalky area to the total grain area, defined as chalkiness size, and the average value was adopted. DEC was calculated as PGWC × chalkiness size × 100%.

### 4.7. Data Analysis

Raw data were processed using Microsoft Excel 2016. Statistica 5.5 [35] was used to perform a *t*-test for comparing trait differences between improved lines and EXSM, as well as between hybrids derived from improved lines and those from EXSM when crossed with the same male sterile lines.

## 5. Conclusions

In this study, MAS combined with backcross breeding was adopted to introgress the long-grain allele of grain shape gene *GL7*/*GW7* into EXSM, and two improved long-grain lines (EXSM-IL1 and EXSM-IL2) were developed. Their background recovery rates were 96.70% and 97.50%, respectively. Their grain shapes largely improved, and grain yield did not change. The hybrids derived from the two improved lines exhibited improved grain shape but no improvement in grain yield. Each of the two improved lines carried 41 favorable alleles associated with vital traits.

## Figures and Tables

**Table 1:** Yield-related traits of the two improved lines.

Trait	EXSM	EXSM-IL1	EXSM-IL2
TGD (d)	131.65 ± 1.09	122.55 ± 2.76 **	123.50 ± 3.51 **
PH (cm)	117.20 ± 2.63	116.05 ± 3.27	116.35 ± 3.33
PN	14.45 ± 3.68	13.75 ± 2.53	11.55 ± 2.48 **
PL (cm)	26.96 ± 0.86	28.05 ± 1.13 **	28.21 ± 1.43 **
PBN	13.73 ± 0.81	13.49 ± 0.89	13.22 ± 0.59 *
SN	168.93 ± 41.31	209.59 ± 42.35 **	179.48 ± 51.18
GN	129.67 ± 32.34	165.38 ± 35.11 **	117.55 ± 31.85
SSR (%)	77.75 ± 3.20	73.15 ± 4.37 **	69.60 ± 5.39 **
KGW (g)	21.55 ± 0.74	19.71 ± 0.58 **	20.54 ± 0.85 **
GY (g)	40.39 ± 8.27	42.36 ± 11.33	36.06 ± 7.79 **

* and ** represent significance level of *p* < 0.05 and 0.01, respectively.

**Table 2:** Yield, appearance quality and milling quality performances of the hybrid combinations of three male sterile lines and the two improved lines.

Trait	Q211S/EXSM	Q211S/EXSM-IL1	Q211S/EXSM-IL2	Z9311S/EXSM	Z9311S/EXSM-IL1	Z9311S/EXSM-IL2	Q9311A/EXSM	Q9311A/EXSM-IL1	Q9311A/EXSM-IL2
TGD (d)	134.67 ± 1.53	132.33 ± 0.58 *	131.33 ± 1.15 *	133.33 ± 1.15	127.67 ± 0.58 **	128.00 ± 1.14 **	136.67 ± 0.58	129.00 ± 1.21 **	128.67 ± 1.53 **
PH (cm)	133.33 ± 1.53	128.67 ± 2.08 *	127.67 ± 1.53 *	135.67 ± 1.53	127.33 ± 7.37	134.00 ± 1.01	124.67 ± 0.58	121.00 ± 1.03 **	124.00 ± 1.02
PN	16.40 ± 2.07	16.20 ± 4.02	16.80 ± 4.15	17.20 ± 1.30	16.80 ± 5.81	15.80 ± 1.92	12.40 ± 1.14	12.20 ± 3.11	11.40 ± 4.39
PL (cm)	27.44 ± 0.83	27.43 ± 0.70	27.79 ± 0.64	29.25 ± 1.73	30.42 ± 1.05 *	29.36 ± 1.96	28.56 ± 1.02	27.25 ± 1.95	26.87 ± 2.31 *
PBN	15.53 ± 0.83	14.67 ± 1.23 *	15.40 ± 1.12	15.07 ± 1.28	14.53 ± 1.19	14.80 ± 0.77	15.20 ± 1.01	13.73 ± 0.80 **	14.27 ± 0.88
SN	228.89 ± 62.51	215.15 ± 30.94	211.47 ± 15.17	152.46 ± 55.22	187.28 ± 36.6	196.90 ± 57.98 *	223.63 ± 56.44	240.44 ± 11.75	231.95 ± 28.20
GN	161.26 ± 38.82	153.83 ± 19.21	148.83 ± 9.54	137.68 ± 37.96	149.98 ± 24.95 *	158.77 ± 37.15 *	157.31 ± 38.53	152.85 ± 8.22	164.24 ± 24.73
SSR (%)	71.24 ± 4.20	71.50 ± 6.51	70.48 ± 3.65	70.68 ± 3.48	70.48 ± 3.65	64.01 ± 3.54 *	70.45 ± 1.59	63.57 ± 1.41 **	70.68 ± 3.48
KGW (g)	25.67 ± 0.04	23.20 ± 0.04 **	23.54 ± 0.05 **	28.12 ± 0.04	26.92 ± 0.04 **	26.54 ± 0.05 **	27.39 ± 0.09	26.57 ± 0.04 *	28.12 ± 0.04
GY (g)	68.91 ± 12.16	67.74 ± 12.72	67.46 ± 15.87	55.24 ± 17.95	61.03 ± 12.80	49.46 ± 16.94	59.15 ± 11.46	50.89 ± 11.53	56.86 ± 12.23
GL (mm)	9.84 ± 0.11	10.17 ± 0.1 **	10.27 ± 0.13 **	9.43 ± 0.12	9.76 ± 0.27 **	9.77 ± 0.21 **	9.69 ± 0.18	10.01 ± 0.14 **	10.17 ± 0.06 **
GW (mm)	2.73 ± 0.10	2.29 ± 0.12 **	2.43 ± 0.07 **	2.70 ± 0.08	2.58 ± 0.11 **	2.59 ± 0.10 **	2.77 ± 0.79	2.29 ± 0.11 **	2.27 ± 0.11 **
LWR	3.61 ± 0.15	4.45 ± 0.25 **	4.22 ± 0.14 **	3.51 ± 0.10	3.76 ± 0.23 **	3.79 ± 0.14 **	3.64 ± 0.55	4.39 ± 0.26 **	4.48 ± 0.22 **
PGWC (%)	27.80 ± 2.17	25.40 ± 1.95	22.00 ± 5.61 *	22.00 ± 5.61	18.80 ± 6.1	17.80 ± 4.38	17.20 ± 1.64	16.60 ± 3.21	13.60 ± 1.52 *
DEC (%)	7.87 ± 0.93	6.44 ± 2.11	6.62 ± 3.35	4.13 ± 0.28	3.86 ± 1.84	3.97 ± 1.21	5.84 ± 1.21	4.96 ± 2.21	4.95 ± 1.16
BRR (%)	78.40 ± 1.27	78.71 ± 0.44	78.35 ± 1.13	80.49 ± 0.96	79.62 ± 0.99	78.35 ± 1.13 *	78.47 ± 0.58	77.80 ± 1.01	79.33 ± 0.55
MRR (%)	73.94 ± 0.94	71.30 ± 0.68 *	70.78 ± 2.29 *	73.94 ± 0.47	72.84 ± 1.17	71.30 ± 0.68 **	73.96 ± 0.88	71.04 ± 1.29 **	71.92 ± 0.99 **
HMRR (%)	55.20 ± 1.39	45.34 ± 4.99	38.94 ± 8.91 **	60.71 ± 6.05	57.06 ± 3.19	59.49 ± 3.18	53.91 ± 1.15	50.53 ± 4.36	47.82 ± 5.21 *

* and ** represent significance levels of *p* < 0.05 and 0.01, respectively. The hybrid combinations derived from improved lines and male sterile lines were compared with the hybrid combinations derived from EXSM and the same male sterile lines.

**Table 3:** Favorable genes contained in the two improved lines.

Category	Gene	Function of Favorable Alleles	EXSM	ZS97-P	EXSM-IL1	EXSM-IL2
Yield	*gn1a*	Increase SN	√	√	√	√
	*NOG1*	Increase SN	√		√	√
	*GL3.2*	Increase GL	√		√	√
	*GNP1*	Increase SN	√	√	√	√
	*gs3*	Increase grain size	√	√	√	√
	*gs5*	Decrease GW and increase the LWR	√	√	√	√
	*CYP704A3*	Increase GL	√	√	√	√
	*BG2*	Increase grain size	√			√
	*GLW7*	Increase grain size	√	√	√	√
	*GW8*	Increase GW	√	√	√	√
Heading Date	*HESO1*/*OsHESO1*	Delay heading	√	√	√	√
	*OsMADS51*	Delay heading	√	√	√	√
	*DTH2*	Delay heading	√	√	√	√
	*DTH3*	Delay heading	√	√	√	√
	*Hd1*	Early heading	√		√	√
	*Ghd7.1*	Early heading		√	√	√
	*RCN1*	Delay heading	√		√	√
Plant Architecture	*SMG11*	Decrease tillering angle	√		√	√
	*D61*/*OsBRI1*	Increase flag leaf angle	√	√	√	√
	*TAC3*	Decrease tillering angle	√		√	√
	*tig1*	Decrease tillering angle	√	√	√	√
Fertility	*Rf3*	Restore fertility	√		√	√
Eating Quality	*badh2*	Produce fragrance	√		√	√
	*Wx*	Amylose content	*Wx^b^*		*Wx^b^*	*Wx^b^*
Biotic Resistance	*Bph3*	Increase brown planthopper resistance	√		√	√
	*PiPR1*	Increase blast resistance	√	√	√	√
	*Pi2*	Increase blast resistance	√		√	√
	*Pi5*	Increase blast resistance	√		√	
	*Xa26*/*Xa3*	Increase bacterial resistance	√		√	√
	*Pia*	Increase blast resistance	√		√	√
	*Xa21*	Increase bacterial resistance	√		√	√
	*Pita*	Increase blast resistance	√		√	√
	*Pid2*	Increase blast resistance	√	√	√	√
Abiotic Resistance	*SKC1*	Increase salt tolerance	√		√	√
	*NRAT1*	Increase aluminum toxicity tolerance	√	√	√	√
	*qLTG3-1*	Increase germination rate at low temperature	√	√	√	√
	*BET1*	Increase boron toxicity resistance	√	√	√	√
	*OsHKT1;1*	Increase Na^+^ content	√		√	√
	*DRO1*	Increase drought tolerance	√	√	√	√
Other	*PSR1*	Increase ratooning ability	√	√	√	√
	*OsHKT2;1*	Increase K^+^ use efficiency	√	√	√	√
	*NRT1.1B*	Increase N use efficiency	√	√	√	√

Note: √ represents the line carries the favorable allele of the gene.

## Data Availability

The original contributions presented in the study are included in the article. Further inquiries can be directed to the corresponding author.

## Abbreviations

The following abbreviations are used in this manuscript:

EXSM	Exiangsimiao
ZS97-P	ZS97 improved line
Q211S	Quan 211S
Z9311S	Zhen 9311S
Q9311A	Quan 9311A
MAS	marker-assisted selection
PS	phenotype selection
SNP	single-nucleotide polymorphism
KASP	Kompetitive Allele Specific
GL	grain length
LWR	length-to-width ratio
GW	grain width
PGWC	percentage of grains with chalkiness
DEC	chalkiness degree
BRR	Brown rice rate
MRR	milled rice rate
HMRR	head milled rice rate
TGD	total growth duration
PH	plant height
PN	panicle number
PL	panicle length
PBN	primary branch number
SN	spikelet number per panicle
GN	grain number per panicle
SSR	seed setting ratio
KGW	1000-grain weight
GY	yield per plant
